# Somatostatin receptor subtype 2 in high-grade gliomas: PET/CT with ^68^Ga-DOTA-peptides, correlation to prognostic markers, and implications for targeted radiotherapy

**DOI:** 10.1186/s13550-015-0106-2

**Published:** 2015-04-22

**Authors:** Aida Kiviniemi, Maria Gardberg, Janek Frantzén, Marko Pesola, Ville Vuorinen, Riitta Parkkola, Tuula Tolvanen, Sami Suilamo, Jarkko Johansson, Pauliina Luoto, Jukka Kemppainen, Anne Roivainen, Heikki Minn

**Affiliations:** Turku PET Centre, Turku University Hospital, University of Turku, Kiinamyllynkatu 4-8, 20521 Turku, Finland; Department of Radiology, Medical Imaging Centre of Southwest Finland, Turku University Hospital, Kiinamyllynkatu 4-8, 20521 Turku, Finland; Department of Pathology, Turku University Hospital, Kiinamyllynkatu 4-8, 20521 Turku, Finland; Department of Neurosurgery, Turku University Hospital, Hämeentie 11, 20521 Turku, Finland; Department of Oncology and Radiotherapy, Turku University Hospital, Hämeentie 11, 20521 Turku, Finland; Turku Center for Disease Modeling, University of Turku, Kiinamyllynkatu 10, 20520 Turku, Finland

**Keywords:** High-grade glioma, Somatostatin receptor, PET/CT, ^68^Ga-DOTANOC, *IDH1* mutation

## Abstract

**Background:**

High-grade gliomas (HGGs) express somatostatin receptors (SSTR), rendering them candidates for peptide receptor radionuclide therapy (PRRT). Our purpose was to evaluate the potential of ^68^Ga-DOTA-1-Nal^3^-octreotide (^68^Ga-DOTANOC) or ^68^Ga-DOTA-Tyr^3^-octreotide (^68^Ga-DOTATOC) to target SSTR subtype 2 (SSTR_2_) in HGGs, and to study the association between SSTR_2_ expression and established biomarkers.

**Methods:**

Twenty-seven patients (mean age 52 years) with primary or recurrent HGG prospectively underwent ^68^Ga-DOTA-peptide positron emission tomography/computed tomography (PET/CT) before resection. Maximum standardized uptake values (SUVmax) and receptor binding potential (BP) were calculated on PET/CT and disruption of blood–brain barrier (BBB) from contrast-enhanced T1-weighted magnetic resonance imaging (MRI-T1-Gad). Tumor volume concordance between PET and MRI-T1-Gad was assessed by Dice similarity coefficient (DC) and correlation by Spearman’s rank. Immunohistochemically determined SSTR_2_ status was compared to receptor imaging findings, prognostic biomarkers, and survival with Kruskal-Wallis, Pearson chi-square, and multivariate Cox regression, respectively.

**Results:**

All 19 HGGs with disrupted BBB demonstrated tracer uptake. Tumor SUVmax (2.25 ± 1.33) correlated with MRI-T1-Gad (*r* = 0.713, *P* = 0.001) although DC 0.41 ± 0.19 suggested limited concordance. SSTR_2_ immunohistochemistry was regarded as positive in nine HGGs (32%) but no correlation with SUVmax or BP was found. By contrast, SSTR_2_ expression was associated with *IDH1* mutation (*P* = 0.007), oligodendroglioma component (*P* = 0.010), lower grade (*P* = 0.005), absence of *EGFR* amplification (*P* = 0.021), and longer progression-free survival (HR 0.161, CI 0.037 to 0.704, *P* = 0.015).

**Conclusions:**

In HGGs, uptake of ^68^Ga-DOTA-peptides is associated with disrupted BBB and cannot be predicted by SSTR_2_ immunohistochemistry. Thus, PET/CT shows limited value to detect HGGs suitable for PRRT. However, high SSTR_2_ expression portends favorable outcome along with established biomarkers such as *IDH1* mutation.

**Trial registration:**

ClinicalTrials.gov NCT01460706

## Background

High-grade gliomas (HGGs) are the most common primary malignant brain tumors with a dismal prognosis [1]. Few treatment options are available in HGGs at the time of recurrence which typically is local. Beneficial response to peptide receptor radionuclide therapy (PRRT) targeting somatostatin receptors has been observed in pilot studies including both low-grade (WHO grade II) and high-grade (WHO grade III and IV) gliomas [2,3]. Additionally, Heute et al. reported encouraging results with recurrent glioblastomas locally treated with ^90^Y-DOTATOC, with a complete remission in one patient [4]. Therefore, PRRT remains an appealing treatment modality especially in recurrent HGGs. However, PRRT is technically demanding and careful selection of patients who are likely to respond is therefore required.

Somatostatin receptor (SSTR) expression in HGGs is controversial. While autoradiographic binding studies have shown no SSTR expression in glioblastomas, immunohistochemistry (IHC) and western blot have detected increased expression of subtypes SSTR_1_, SSTR_2_, and SSTR_3_ [5,6]. Therefore, an *in vivo* method to identify SSTR_2_ expression, the most abundant subtype, in patients who are candidates for PRRT is required. While scintigraphic studies have shown variable uptake of octreotide analogs in gliomas, we are not aware of prospective studies on ^68^Ga-DOTANOC or ^68^Ga-DOTATOC uptake in gliomas with positron emission tomography (PET), which has superior sensitivity compared to scintigraphy in detecting SSTR_2_-positive neuroendocrine tumors [7,8].

Our understanding on the genetic alterations in gliomas has led to the recognition of several molecular subtypes with distinct clinical, prognostic, and imaging characteristics [9,10]. *IDH1* mutation, *MGMT* promoter methylation, and 1p/19q co-deletion are the three molecular markers included today in routine assessment of gliomas due to their diagnostic, prognostic, or predictive value [11]. An association between these molecular biomarkers and SSTR_2_ expression in HGGs, however, has not been studied before.

We aimed to prospectively study the potential of ^68^Ga-DOTANOC and ^68^Ga-DOTATOC to target SSTR_2_ in HGGs and to detect HGGs suitable for PRRT *in vivo*. We hypothesized that SSTR_2_ expression in HGGs could be quantified with dynamic positron emission tomography/computed tomography (PET/CT) using ^68^Ga-DOTA-peptides. Also, our aim was to characterize SSTR_2_ expression in HGGs in relation to prognostic markers such as *IDH1* mutation.

## Methods

Twenty-seven patients with radiologically suspected primary (*n* = 17) or recurrent (*n* = 10) HGG scheduled for tumor resection between 2011 and 2013 were prospectively enrolled (mean age 52 years; 17 women and 10 men). PET/CT was performed prior to surgical resection with a mean interval of 19 days. Additionally, one patient with primary HGG was included in SSTR_2_ IHC analysis without performing PET/CT. ^68^Ga-DOTANOC replaced ^68^Ga-DOTATOC as the tracer in practice after first three patients due to poor availability of the DOTATOC precursor. Patient characteristics are presented in Table 1. The study was approved by the Ethics Committee of the Hospital District of Southwest Finland and the Finnish Medicines Agency. All patients gave written informed consent before participation. The study has been registered at ClinicalTrials.gov (NCT01460706).

**Table 1:** **Clinical data, imaging abnormalities, and SSTR**_**2**_ **expression**

**Clinical data**									**Imaging abnormalities**				**SSTR**_**2**_		
**Pt no**	**Age/sex**	**Stage**	**Previous therapy**	**Gr**	**Dg**	**PFS (months)**	**OS (months)**	**Follow-up (months)**	***V***_**T1-Gad**_ **(cm** ^**3**^ **)**	**DOTA**	**SUVmax**	**BP**	**C**	**H**	**L**
1	49/F	Primary		III	A			29.4	0.57	TOC	0.79	−0.26	0	2	B
2	62/F	Primary		III	A			14.4	1.71	NOC	1.22^b^	NA	0	2	B
3	44/F	Primary		III	A			21.0	0	NOC	−^c^	−	1	3	B
4	49/M	Primary		III	A			13.0	0	NOC	−	−	3	3	C
5	57/F	Primary		III	A	10.4		12.5	0.16	NOC	0.69	−0.47	0	0	−
6	18/M	Primary		III	A	10.9		11.8	0	NOC	−	−	0	3	B
7	49/F	Primary		III	A			10.7	0.81	NOC	0.69 ^d^	NA	1	3	C
8	32/F	Primary		III	A	8.4		13.8	0	X	X	X	0	1	C
9	54/F	Primary		III	O	13.6		27.7	0.03	NOC	−	−	3	3	B
10	60/M	Primary		III	OA			21.6	0	NOC	−	−	3	3	B
11	71/M	Primary		IV	GBMO	0	1.2		1.91	NOC	3.07	1.41	0	3	M
12	37/M	Primary		IV	GBMO			21.0	4.32	NOC	0.99	−0.62	3	3	B
13	35/F	Primary		IV	GBM	0	13.7		1.27	TOC	0.65	0.39	0	1	C
14	63/F	Primary		IV	GBM	24.0		24.1	12.2	NOC	2.21	0.98	0	0	−
15	62/F	Primary		IV	GBM	0	7.3		11.5	NOC	3.01	1.26	0	0	−
16	67/F	Primary		IV	GBM	0	4.4		14.8	NOC	1.62	0.36	0	1	B
17	70/M	Primary		IV	GBM	0	1.0		21.5	NOC	2.85	1.80	0	3	M
18	76/F	Primary		IV	GS	0	0.9		34.7	NOC	3.73	1.75	0	3	M
19	28/M	Recurrent	S	III	A	81.2		106.1	0	NOC	−	−	1	3	B
20	51/M	Recurrent	S, RT	III	O	60.9		253.7	0	NOC	−	−	3	3	B
21	42/F	Recurrent	S, S, RT, S	III	OA	24.3	99.5		0	NOC	−	−	3	3	C
22	46/M	Recurrent	S, RT, C	III	OA	39.0	100.4		28.2	NOC	5.68	3.33	1	1	C
23	42/F	Recurrent	S, CRT	IV	GBM	24.0	37.1		0.04	TOC	0.46	−0.34	0	3	B
24	68/F	Recurrent	S, CRT	IV	GBM	23.1	26.3		27.9	NOC	2.40	1.57	0	0	−
25	64/F	Recurrent	S, CRT	IV	GBM	34.2	38.2		12.8	NOC	2.70	1.07	0	3	B
26	42/F	Recurrent	S, CRT	IV	GBM	5.5	13.6		28.0	NOC	2.44	2.28	0	3	B
27	61/F	Recurrent	S, RT	IV	sGBM	16.1	21.3		2.59	NOC	1.98	0.64	0	2	C
28	57/M	Recurrent	S, CRT	IV	GBM	11.0	27.5		3.03^a^	NOC	3.26	2.33	0	3	B

Pt no, patient number; F, female; M, male; Gr, grade; Dg, diagnosis; A, astrocytoma; O, oligodendroglioma; OA, oligoastrocytoma; GBMO, glioblastoma with oligodendroglioma component; GBM, glioblastoma; sGBM, secondary GBM; GS, gliosarcoma; S, surgery; RT, radiotherapy; C, chemotherapy (temozolomide); CRT, chemoradiotherapy with adjuvant temozolomide; Dx, dexamethasone; TOC, ^68^Ga-DOTATOC; NOC, ^68^Ga-DOTANOC; −, no tracer uptake; X, no PET performed; NA, not applicable; C, intensity of most common staining (0 to 3); H, highest staining intensity (0 to 3); L, location of staining; C, cytoplasmic; M, membranous; B, both. ^a^No MRI (cardiac pacemaker), tumor volume defined from contrast-enhanced CT; ^b^static PET 28 to 58 min post-injection (mild claustrophobia); ^c^dynamic PET discontinued at 53 min post-injection (dyspnea); ^d^dynamic PET discontinued at 36 min post-injection (numbness of the arm).

### PET/CT with ^68^Ga-DOTA-peptides and MRI

^68^Ga-DOTA-peptides were synthesized as previously described [12]. Radiochemical purity and specific activity (mean) was 99.9% and 31.5 GBq/μmol for ^68^Ga-DOTANOC, and 96.5% and 26.8 GBq/μmol for ^68^Ga-DOTATOC, respectively. PET/CT of the brain was performed using a GE Discovery VCT PET/CT Scanner (General Electric Medical Systems, Pewaukee, WI, USA). Dynamic PET was acquired over 60 min (8 × 15-, 6 × 30-, 5 × 180-, 4 × 300-, and 2 × 600-s frames) after intravenously injected ^68^Ga-DOTANOC (123 MBq, median) or ^68^Ga-DOTATOC (130 MBq, median). Low-dose CT (120 kV, 10 to 95 mA, noise index 25, slice thickness 3.75 mm) was used for attenuation correction. PET images were reconstructed (256 × 256 matrix, OSEM3D, 3 iterations, 28 subsets, 4.8-mm Hanning postfilter) yielding a pixel size of 1.4 mm. A duration of 60 min was determined after 90-min dynamic PET performed to one patient (no. 13) and 5-min static scans performed to three patients (nos. 11, 12, and 23) 96 min post-injection confirmed that tumor uptake did not increase after 60 min.

Clinical preoperative MRI nearest in time to PET scan (mean interval 14 days) was employed, and post-contrast T1-weighted images (MRI-T1-Gad) were used to define the tumor volume with contrast enhancement. MRI scanners and parameters used are listed in Online Resource 1.

### Image evaluation

Analyses were performed using in-house developed software (Carimas 2.7; http://www.turkupetcentre.fi/carimas/). PET and MRI-T1-Gad images were co-registered, and spherical volumes of interest (VOIs) were manually placed over the tumor area with maximum activity. VOIs for contralateral normal brain white matter, nasal mucosa, pituitary gland, and skin of the occiput were also defined, and maximum standardized uptake values (SUVmax) were calculated (SUV = [Tissue radioactivity concentration (Bq/ml) × Body weight (g)]/Injected dose (Bq)). Tumor mean SUVmax 30 to 60 min post-injection was used for further analyses.

Logan plot with skin as a reference tissue was used for tracer kinetic modeling [13]. We used 5 min as the starting point for linear regression model that was optimized against the normalized tumor and reference tissue time-activity curves (TAC), with the slope being the distribution volume ratio (DVR). Binding potential (BP) corresponds to the density of available receptors and is calculated as BP = DVR − 1.

For comparison of tumor volume abnormalities in PET and MRI-T1-Gad, the images were co-registered by automatic fusion using iPlan RT Treatment Planning Software (Brainlab, Munich, Germany). Tumor PET volumes were contoured with a threshold of 40% SUVmax (*V*_PET_). Contrast-enhanced tumor volume in MRI-T1-Gad (*V*_T1-Gad_) was delineated by thresholding the enhancing tumor volume on visual basis and then manually subtracting the hyperintense volume on precontrast T1-weighted images. Overlapping volumes between *V*_PET_ and *V*_T1-Gad_ were determined, and Dice similarity coefficient (DC = [2 × Intersection]/[*V*_PET_ + *V*_T1-Gad_]) was calculated. A DC value of 1 indicates perfect similarity between the volumes, while a value of 0 indicates no similarity. Patient no. 5 was excluded from analysis due to low SUVmax following failure in PET volume delineation.

### Immunohistochemistry and molecular markers

Formalin-fixed paraffin-embedded tumor tissues were sectioned at 3 μm and used for analyses. Antibodies for IHC are listed in Table 2. *MGMT* promoter methylation was studied by pyrosequencing [14], 1p/19q co-deletion by fluorescent *in situ* hybridization, and *EGFR* amplification by silver *in situ* hybridization [15].

**Table 2:** **Primary antibodies and methods used for immunohistochemistry**

**Antibody**	**Clone**	**Manufacturer**	**Method**	**Detection**
SSTR_2_	UMB-1	Abcam, Cambridge, UK	Ventana Benchmark XT Autostainer (Ventana Medical Systems, Strasbourg, France)	*ultra*VIEW Universal Detection Kit (Ventana, Strasbourg, France)
SSTR_3_	Rabbit polyclonal (ab28680)	Abcam, Cambridge, UK	Labvision Autostainer (Thermo Scientific Inc, Kalamazoo, MI)	BrightVision Detection Kit (Immunologic, Duiven, the Netherlands)
SSTR_5_	Rabbit polyclonal (AB5681)	Millipore, Billerica, MA	Labvision Autostainer	BrightVision Detection Kit
EGFR	5B7	Ventana, Strasbourg, France	Ventana Benchmark XT Autostainer	*ultra*VIEW Universal Detection Kit
IDH1 R132H	H09	Dianova, Hamburg, Germany	Ventana Benchmark XT Autostainer	*ultra*VIEW Universal Detection Kit
CD68	PG-M1	Dako, Glostrup, Denmark	Ventana Benchmark XT Autostainer^a^	*ultra*VIEW Universal Detection Kit + Universal Alkaline Phosphatase Red Detection Kit (Ventana)^a^
Ki67	30-9	Ventana, Strasbourg, France	Ventana Benchmark XT Autostainer	*ultra*VIEW Universal Detection kit

^a^SSTR_2_ + CD68 double staining.

SSTR_2_ IHC was scored first independently by an experienced neuropathologist (MG) and finally in consensus with a second observer (AK) both blinded to PET data. Double staining with UMB1 + CD68 was used to exclude SSTR_2_ staining in microglia and macrophages and to evaluate the number of these cells in the tumor specimen by counting all CD68-positive cells and tumor cells in one representative high-power field. In diffuse gliomas, HE staining and IHC for IDH1 mutation assisted in localizing tumor cells. Three scoring parameters were reported for SSTR_2_ staining: the highest (minimum 10% of tumor area), the most common staining intensity, and the localization of staining (membranous, cytoplasmic, or both). Staining intensities were classified as follows: 0 (negative), 1 (weak), 2 (moderate), and 3 (strong). The same scoring parameters were used for EGFR IHC. SSTR_2_ IHC was further categorized as positive if the sum of the highest and most common staining intensities was ≥4 and negative if the sum was <4.

### Statistical analysis

Data are presented as mean ± SD. Pearson or Spearman’s rank correlations were used between tracer uptake parameters and *V*_T1-Gad_. *V*_PET_ and *V*_T1-Gad_ were compared with Mann–Whitney *U* test. The association between SSTR_2_ status and molecular markers was studied with Pearson chi-square analysis by cross-tabulations, and difference in Ki67 with independent samples *t* test. SSTR_2_ IHC and tracer uptake were compared with Kruskal-Wallis. Kaplan-Meier curves with log-rank test and univariate Cox regression were used to analyze progression-free survival (PFS) and overall survival (OS). Multivariate Cox regression (backward Wald) was performed for PFS with SSTR_2_ status and tumor grade as variables. PFS was defined as the time from the first surgical resection (also for recurrent HGGs) to the first tumor progression in MRI, deterioration in clinical symptoms, or end of follow-up. OS was defined as the time from the first surgical resection to death or end of follow-up. Two-tailed *P* values <0.05 were regarded significant. Statistical analyses were conducted using SPSS 21 for Mac (SPSS Inc., Chicago, IL, USA).

## Results

**Figure 1:**
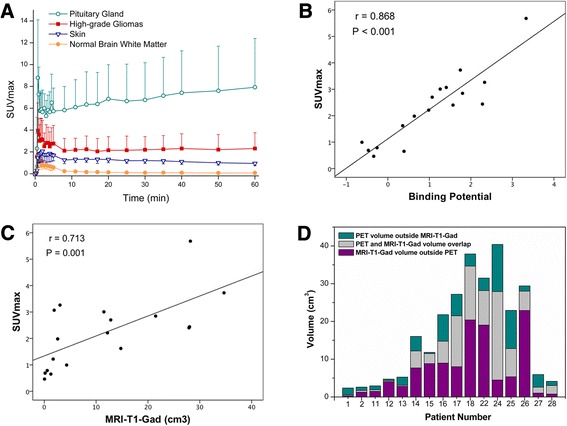
^68^Ga-DOTA-peptide uptake in PET and its comparison to enhancing tumor volume in MRI-T1-Gad. Time-activity curves show higher ^68^Ga-DOTA-peptide uptake in HGGs compared to skin but distinctly lower uptake when compared to pituitary gland **(A)**. Tumor SUVmax at 30 to 60 min post-injection correlates to receptor binding potential **(B)** and to enhancing tumor volume in MRI-T1-Gad **(C)**. However, PET and MRI-T1-Gad tumor volumes show apparent discordance in individual patients **(D)** (corresponding to patient numbers in Table 1).

All 19 HGGs with uptake demonstrated disrupted blood-brain barrier (BBB) in MRI-T1-Gad whereas no uptake was detected with intact BBB. Rapid peak in tumor uptake was followed by gradual decline reaching a plateau in 15 min (Figure 1A). Tumor SUVmax 30 to 60 min post-injection was 2.25 ± 1.33 (range 0.46 to 5.68), and it correlated with MRI-T1-Gad (*r* = 0.713, *P* = 0.001; Figure 1C). Tumor-to-skin SUVmax ratio was 2.12 ± 1.11.

Regression line of the Logan plot fitted to each data set successfully. BP in HGGs with uptake was 1.03 ± 1.11, and it correlated with tumor SUVmax (*r* = 0.868, *P* < 0.001; Figure 1B) and tumor-to-skin SUVmax ratio (*r* = 0.956, *P* < 0.001), implicating that SUVmax in this setting is a good marker for specific receptor binding. Negative BP values indicating lower tumor concentration of available receptors compared to reference tissue were found in HGGs with SUVmax <1.0.

Difference in *V*_PET_ (9.3 ± 9.5 cm^3^) and *V*_T1-Gad_ (12.9 ± 11.7 cm^3^) was nonsignificant (*P* = 0.559). [*V*_PET_]/[V_T1-Gad_] tumor volume proportion was 1.05 ± 0.87. Dice similarity coefficient between *V*_PET_ and *V*_T1-Gad_ was 0.41 ± 0.19. Concordance and discordance between PET and MRI-T1-Gad tumor volumes in individual patients are demonstrated in Figure 1D.

### SSTR IHC and correlation to PET

**Figure 2:**
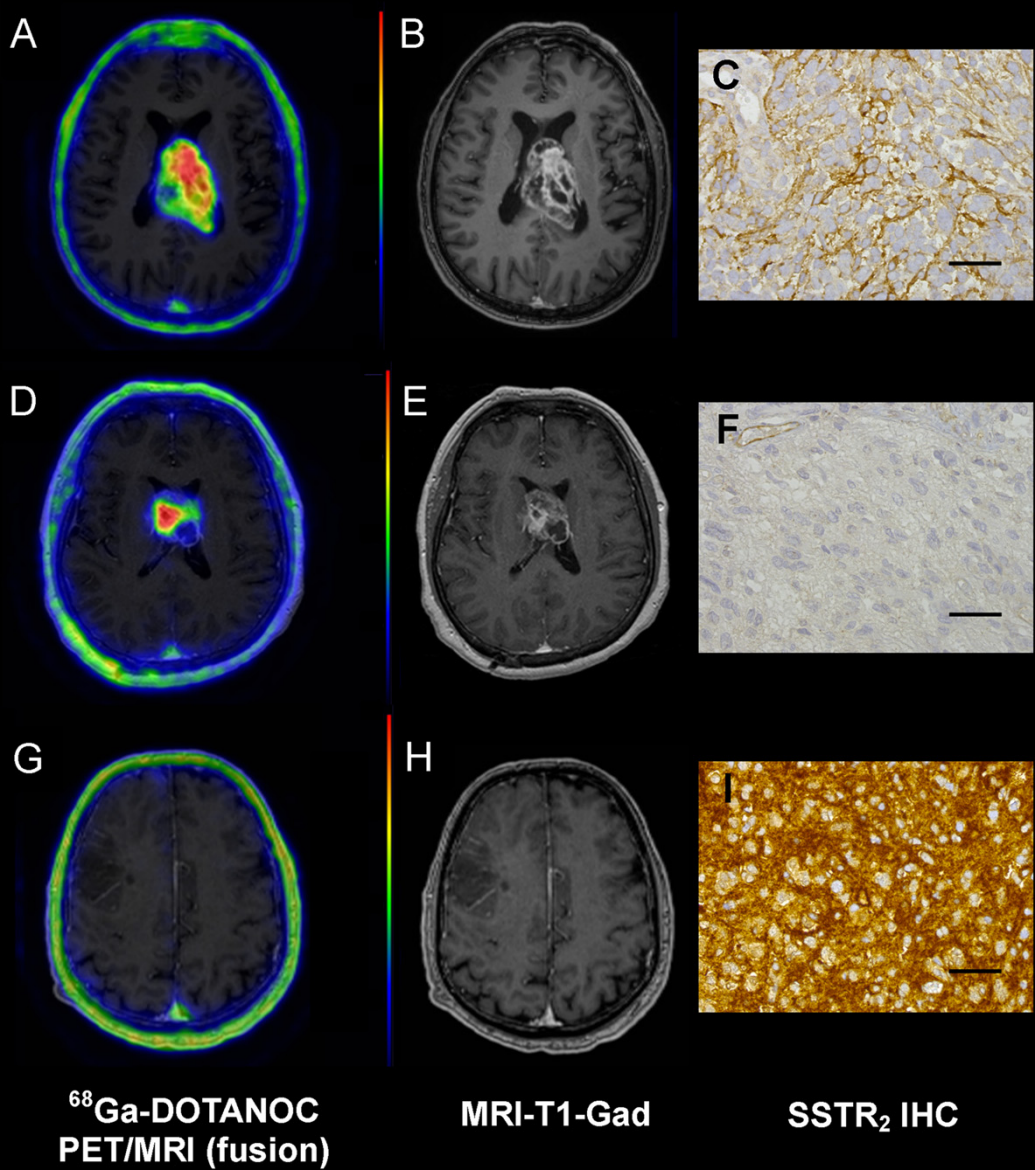
^68^Ga-DOTA-peptide uptake in high-grade gliomas does not correspond to SSTR_2_ immunohistochemistry. Axial fused PET/MR images 30 to 60 min post-injection, corresponding contrast-enhanced T1-weighted MR images, and tumor SSTR_2_ IHC from three different patients. Primary glioblastoma (patient no. 17) presents ^68^Ga-DOTANOC uptake **(A)** and contrast-enhancement in MRI-T1-Gad **(B)**. Patchy areas of positive SSTR_2_ staining were observed **(C)**. Another primary glioblastoma (patient no. 15) also shows ^68^Ga-DOTANOC uptake **(D)** and contrast enhancement **(E)**. However, SSTR_2_ IHC was negative **(F)**. Primary oligoastrocytoma (patient no. 10) represents no ^68^Ga-DOTANOC uptake **(G)** and no contrast enhancement **(H)**, but high SSTR_2_ expression in IHC was detected **(I)**. Color scale in PET images is set to maximum (red) 10,000 Bq/ml and minimum (blue) 0 Bq/ml. Bar = 50 μm.

SSTR_2_ expression in HGGs showed considerable variation (Table 1). When dichotomized, SSTR_2_ IHC was regarded as positive in 8 anaplastic gliomas and 1 GBMO (32%) and as negative in 6 anaplastic gliomas and 13 glioblastomas (GBMs) (68%). However, no correlation with SUVmax or BP was found (Figure 2). In fact, 7 out of 8 HGGs with no ^68^Ga-DOTA-peptide uptake were classified as SSTR_2_ positive, while 17 out of 19 HGGs with tracer uptake were classified as SSTR_2_ negative. Since most HGGs demonstrated patchy areas of SSTR_2_ reactivity, though regarded as SSTR_2_ negative, we studied whether SUVmax or BP associates to the highest staining intensity observed. However, no association was found (SUVmax *P* = 0.613; BP *P* = 0.655). SUVmax for HGGs with membranous SSTR_2_ staining (3.21 ± 0.46) was higher compared to that for HGGs demonstrating both membranous and cytoplasmic staining (1.69 ± 1.01); however, the difference was not significant (*P* = 0.082). SSTR_3_ immunoreactivity was detected in cells bordering necrosis in seven GBM samples. One glioma (no. 2) displayed positive SSTR_3_ staining in approximately 30% of tumor cells. SSTR_5_ expression was not detected in any of the tumor specimens studied. SSTR_2_ staining in microglia and macrophages was excluded from IHC scoring with double staining. Due to intense CD68 reactivity, however, the SSTR_2_ expression in microglia and macrophages was not assessable. The distribution and number of microglia and macrophages were heterogeneous. Within the cellular part of the tumor, variable diffuse infiltration was observed, whereas dense clusters of microglia and macrophages were detected along the necrosis border in GBMs. The average number of microglia and macrophages in anaplastic gliomas ranged from 0% to 4%, to 20% to 24% (median 5% to 9%) and in GBMs from 10% to 14%, to 35% to 39% (median 25% to 29%).

### SSTR_2_ IHC, molecular markers, and survival

Positive SSTR_2_ IHC corresponded with *IDH1* mutation (*P* = 0.007), lower tumor grade (*P* = 0.005), and oligodendroglioma component (*P* = 0.010) as presented in Table 3. Furthermore, Ki67 was significantly lower in SSTR_2_-positive HGGs (14.44 ± 10.50) compared to SSTR_2_-negative HGGs (41.05 ± 25.60; *P* = 0.001). Association between SSTR_2_ IHC and *MGMT* promoter methylation was not significant (*P* = 0.080), but it is notable that all SSTR_2_-positive HGGs contained *MGMT* promoter methylation, whereas all unmethylated HGGs were SSTR_2_ negative. Positive SSTR_2_ IHC was related to the absence of *EGFR* amplification (*P* = 0.021). However, no dependence was found between SSTR_2_ and EGFR IHC.

**Table 3:** **Crosstabs on SSTR**_**2**_ **immunohistochemistry (IHC) against molecular markers, histological type, and tumor grade**

	**SSTR**_**2**_ **IHC**		
	**Negative**	**Positive**	**P value**
	***n*** **(%)**	***n*** **(%)**	
*IDH1* mutation			
Yes	3 (33.3)	6 (66.7)	0.007
No	16 (84.2)	3 (15.8)	
1p/19q co-deletion			
Yes	2 (50.0)	2 (50.0)	0.409
No	17 (70.8)	7 (29.2)	
*EGFR* amplification			
Yes	8 (100.0)	0 (0.0)	0.021
No	11 (55.0)	9 (45.0)	
*MGMT* promoter methylation			
Yes	13 (59.1)	9 (40.9)	0.080
No	5 (100.0)	0 (0.0)	
p53 mutation			
Yes	11 (78.6)	3 (21.4)	0.225
No	8 (57.1)	6 (42.9)	
Grade			
III	6 (42.9)	8 (57.1)	0.005
IV	13 (92.9)	1 (7.1)	
Oligodendroglioma component			
Yes	2 (28.6)	5 (71.4)	0.010
No	17 (81.0)	4 (19.0)	

**Figure 3:**
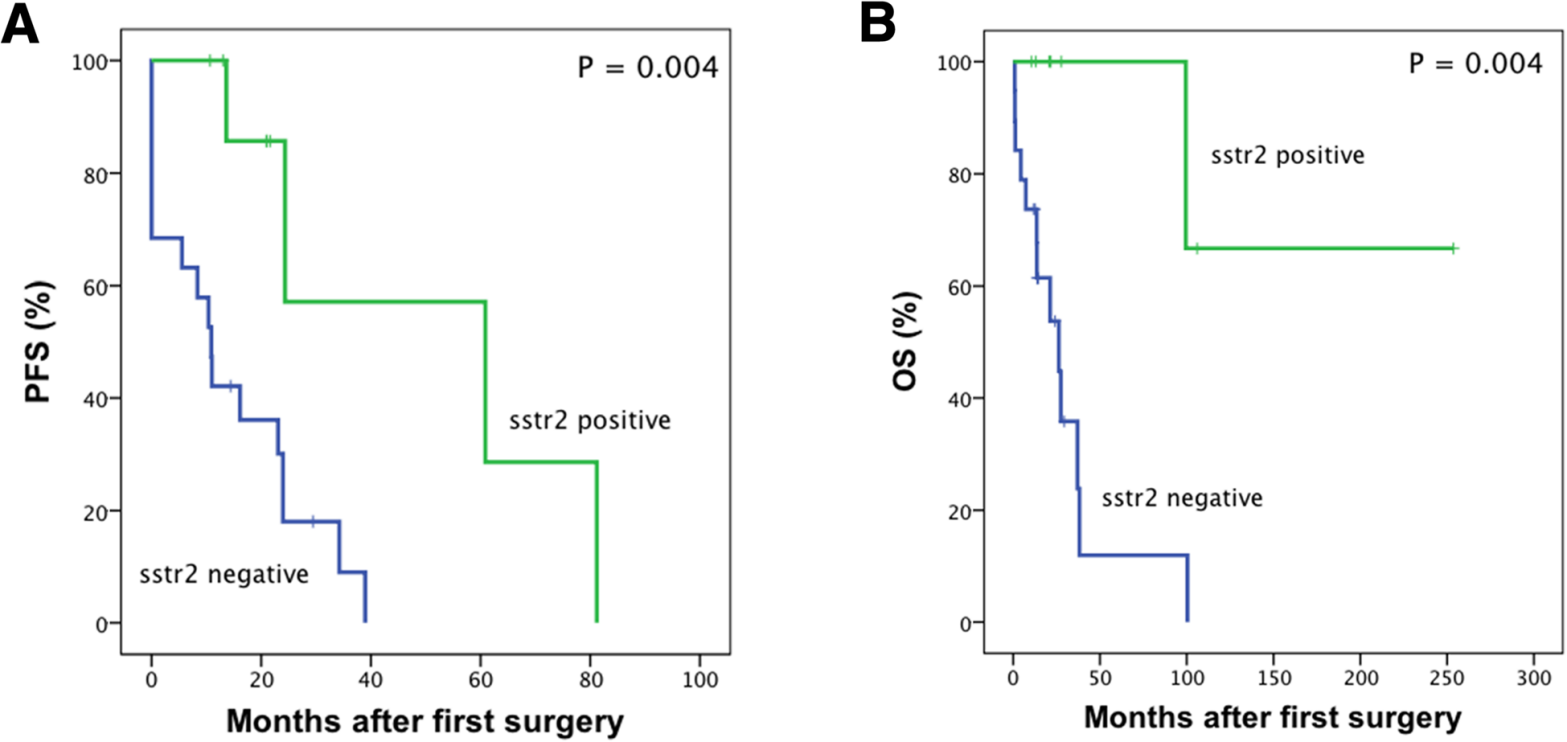
Survival in HGG patients separated by SSTR_2_ status in immunohistochemistry. Kaplan-Meier curves for progression-free survival **(A)** and overall survival **(B)** in HGG patients with positive or negative SSTR_2_ IHC. Censored data are indicated by vertical lines.

Kaplan-Meier curves show the prognostic value of SSTR_2_ status (Figure 3). Median PFS for SSTR_2_-positive and SSTR_2_-negative HGGs was 60.9 versus 10.9 months, respectively. Median OS for SSTR_2_-negative HGGs was 26.3 months. Only one death occurred during follow-up among SSTR_2_-positive HGGs. Within anaplastic gliomas, the median PFS for SSTR_2_-positive and SSTR_2_-negative HGGs was 21.3 versus 12.7 months, respectively.

SSTR_2_ status had prognostic value in HGG patients for both PFS and OS (Table 4). SSTR_2_ expression was an independent prognostic factor for prolonged PFS after adjustment to histological grade (HR 0.161, CI 0.037 to 0.704, *P* = 0.015). Multivariate Cox regression for OS was not applied due to a low number of end points. *IDH1* mutation and *MGMT* promoter methylation were distinct prognostic factors for both PFS (HR 0.312, CI 0.102 to 0.950, *P* = 0.040 and HR 0.234, CI 0.073 to 0.753, *P* = 0.015, respectively) and OS (HR 0.167, CI 0.036 to 0.770, *P* = 0.022 and HR 0.229, CI 0.061 to 0.859, *P* = 0.029, respectively). Improved OS was observed in patients younger than 60 years of age (HR 0.183, CI 0.053 to 0.631, *P* = 0.007).

**Table 4:** **Univariate analysis of prognostic factors for progression-free survival (PFS) and overall survival (OS) in HGG patients**

		**PFS**		**OS**	
	***n*** **(%)**	**HR (95% CI)**	***P*** **value**	**HR (95% CI)**	***P*** **value**
SSTR_2_ IHC					
Negative	19 (67.9)	1		1	
Positive	9 (32.1)	0.161 (0.037 to 0.704)	0.015	0.083 (0.010 to 0.655)	0.018
Age					
> 60	11 (39.3)	1		1	
< 60	17 (60.7)	0.484 (0.190 to 1.233)	0.128	0.183 (0.053 to 0.631)	0.007
Oligodendroglioma component					
No	21 (75.0)	1		1	
Yes	7 (25.0)	0.592 (0.210 to 1.672)	0.322	0.361 (0.094 to 1.380)	0.137
*IDH1*					
Wild type	19 (67.9)	1		1	
Mutated	9 (32.1)	0.312 (0.102 to 0.950)	0.040	0.167 (0.036 to 0.770)	0.022
*MGMT* promoter					
Unmethylated	5 (18.5)	1		1	
Methylated	22 (81.5)	0.234 (0.073 to 0.753)	0.015	0.229 (0.061 to 0.859)	0.029
1p/19q co-deletion					
No	24 (85.7)	1		1	
Yes	4 (14.3)	0.780 (0.244 to 2.496)	0.675	0.390 (0.081 to 1.871)	0.239
*EGFR* amplification					
No	20 (71.4)	1		1	
Yes	8 (28.6)	2.083 (0.819 to 5.297)	0.124	3.131 (0.986 to 9.943)	0.053

Results are expressed as hazard ratio (HR) with 95% confidence interval.

## Discussion

We have demonstrated that ^68^Ga-DOTA-peptide uptake in PET/CT is associated to disrupted BBB but does not correlate to immunohistochemically (IHC) determined SSTR_2_ status, suggesting limited benefit of this approach in defining suitable patients for PRRT. Low tumor SUVmax further implies that the achievable dose with intravenously administered PRRT remains low. The second finding of particular interest was the positive association between SSTR_2_ expression and *IDH1* mutation, oligodendroglioma component, and improved PFS, which indicates that SSTR_2_ expression may become a new biomarker in HGG useful for prognostication and therapeutic decision-making.

Our findings correspond to SSTR scintigraphic studies in low- and high-grade gliomas where ^111^In-DTPA-D-Phe1-octreotide scintigraphy visualized the tumors with disrupted BBB but did not correlate to *in vitro* SSTR autoradiography [16]. The authors reasoned that this discrepancy was due to nonspecific accumulation and trapping of the octreotide molecules. Again, we did not find a correlation between ^68^Ga-DOTA-peptide uptake and SSTR_2_ IHC, which may be related to the heterogeneity of tumor tissue and uncertainties in obtaining the tissue sample from regions with the highest tracer uptake. Our results, however, do not confirm that the uptake is merely due to nonspecific accumulation. First, variation in SUVmax was noticed among similar MRI-T1-Gad volumes (Figure 1C). Second, alteration in BP values (range −0.62 to 3.33) suggests different SSTR_2_ densities in HGGs. Third, the Dice similarity coefficient between tumor volumes in PET and MRI-T1-Gad was rather low (0.41), implicating discordance between these volumes. In other words, tracer uptake was not limited to the area of disrupted BBB but was present in neighboring areas due to diffusion and/or receptor binding. Of note is also low expression of SSTR_3_ and SSTR_5_ which did not explain differences in tracer uptake. By contrast, specific binding is not supported by the time course of tumor ^68^Ga-DOTA-peptide uptake (Figure 1A) as the typical pattern of ligand-binding curve is not present. Thus, nonspecific tracer uptake cannot be excluded since a blocking study requiring another dynamic PET/CT was not performed.

The magnitude of diagnostic radionuclide uptake is not trivial for a successful PRRT. In meningiomas treated with PRRT, SUVmax with ^68^Ga-DOTA-peptides was related to the corresponding ^177^Lu-labeled radionuclide uptake and therapeutic dose [17]. SUVmax in meningiomas ranged from 4.3 to 68.7 with a very low therapeutic dose achieved with the lowest SUVmax. In our study, mean SUVmax was 2.25 with only four GBMs and one anaplastic glioma demonstrating SUVmax >3.0, suggesting an insufficient achievable dose in PRRT. It has to be noted, though, that PRRT in extra-axial meningiomas is intravenously delivered whereas in gliomas invading the brain, the therapeutic radionuclide needs to be locally injected [2-4]. This is of importance especially in HGGs with intact BBB that are SSTR_2_ positive but lack tracer uptake in PET. These tumors may benefit from locally delivered PRRT, which, however, cannot be evaluated by intravenously given ^68^Ga-DOTA-peptides.

To our knowledge, this study demonstrates for the first time that high SSTR_2_ expression in HGG is associated with *IDH1* mutation which is regarded as the most powerful prognostic marker for a favorable outcome compared to *IDH1* wild-type gliomas [18]. It is unclear, however, why *IDH1* mutation affects prognosis and what the interaction with other markers is. Recently, *IDH1* mutation was found to determine the prognostic and predictive value of *MGMT* promoter methylation in anaplastic gliomas [19]. Association with mutant *IDH1* implicates a potential role for SSTR_2_ in the favorable outcome of chemosensitive and radiosensitive HGGs. This is substantiated by the observed trend of correlation between SSTR_2_ expression and *MGMT* promoter methylation.

Previous studies have not detected a clear association between SSTR_2_ expression and oligodendroglioma component. Cervera et al. found increased SSTR_2_ mRNA in oligodendrogliomas but finally concluded that it was due to contamination from normal brain [20]. Our SSTR_2_ scoring was based on IHC with a well-documented monoclonal antibody UMB-1 allowing cellular localization [21]. We found that SSTR_2_ staining was not restricted to oligodendroglial tumor cells but was similarly present in astrocytic cells in mixed oligoastrocytomas. Furthermore, two anaplastic oligodendrogliomas out of the four HGGs with 1p/19q co-deletion, a biomarker strongly linked to oligodendroglial histology [22], also showed the most intensive SSTR_2_ staining. We thus demonstrate an association between SSTR_2_ and oligodendroglioma component but underline that a larger study is necessary to confirm this initial finding.

SSTR_2_ status was an independent prognostic marker for PFS after adjustment to glioma grade. SSTR_2_ expression has previously been associated to improved survival with neuroendocrine tumors [23] and childhood neuroblastomas [24], suggesting a potential role for SSTR_2_ as a tumor suppressor. In fact, this antitumor effect has been demonstrated in experimental pancreatic tumors stably expressing SSTR_2_ [25], or after therapeutic SSTR_2_ gene transfer [26] with inhibited tumor growth. Our results on improved PFS substantiate the anti-oncogenic role of SSTR_2_ in HGGs as well.

Our study confronts limitations. First, two tracers with different affinity profiles for SSTR subtypes were used. However, the highest affinity with both ^68^Ga-DOTANOC and ^68^Ga-DOTATOC is for SSTR_2_ [27]. In addition, our HGG samples demonstrated minimal expression of subtypes SSTR_3_ and SSTR_5_, for which ^68^Ga-DOTANOC possesses higher affinity compared to ^68^Ga-DOTATOC. Also, as ^68^Ga-DOTATOC was applied only in 3 patients and ^68^Ga-DOTANOC in 24 patients, we conclude that most likely this limitation had little effect on the outcome. Second, the number of patients was limited. Our data on established biomarkers, however, was in accordance with current literature giving substantiation also to our SSTR_2_ results. Third, potential alteration in tumor edema affecting volumetric analyses due to differences in steroid dosing during the interval between MRI and PET/CT cannot be excluded. Furthermore, clinical MRI with different scanners and parameters was used for MRI-T1-Gad evaluation.

## Conclusions

We conclude that PET/CT with ^68^Ga-DOTA-peptides provides limited value in identifying patients with SSTR_2_-positive HGGs suitable for PRRT. Our study demonstrates for the first time that SSTR_2_ expression in HGGs is associated with *IDH1* mutation, oligodendroglioma component, and improved PFS. This potential diagnostic and prognostic value for SSTR_2_ expression in HGGs should be confirmed in a larger validation study.

## Abbreviations

A: astrocytoma
BBB: blood–brain barrier
BP: binding potential
DC: Dice similarity coefficient
^68^Ga-DOTANOC: ^68^Ga-DOTA-1-Nal^3^-octreotide
^68^Ga-DOTATOC: ^68^Ga-DOTA-Tyr^3^-octreotide
GBM: glioblastoma
GBMO: glioblastoma with oligodendroglioma component
HGG: high-grade glioma
IHC: immunohistochemistry
MRI-T1-Gad: contrast-enhanced T1-weighted magnetic resonance images
O: oligodendroglioma
OA: oligoastrocytoma
OS: overall survival
PFS: progression-free survival
PRRT: peptide receptor radionuclide therapy
SSTR_2_: somatostatin receptor subtype 2
SUVmax: maximum standardized uptake value
*V*_PET_: tumor volume with ^68^Ga-DOTA-peptide uptake in PET/CT
*V*_T1-Gad_: contrast-enhanced tumor volume in MRI-T1-Gad

## References

[1] Ostrom QT, Bauchet L, Davis FG, Deltour I, Fisher JL, Langer CE (2014). The epidemiology of glioma in adults: a “state of the science” review. Neuro Oncol..

[2] Merlo A, Hausmann O, Wasner M, Steiner P, Otte A, Jermann E (1999). Locoregional regulatory peptide receptor targeting with the diffusible somatostatin analogue 90Y-labeled DOTA0-D-Phe1-Tyr3-octreotide (DOTATOC): a pilot study in human gliomas. Clin Cancer Res..

[3] Schumacher T, Hofer S, Eichhorn K, Wasner M, Zimmerer S, Freitag P (2002). Local injection of the 90Y-labelled peptidic vector DOTATOC to control gliomas of WHO grades II and III: an extended pilot study. Eur J Nucl Med Mol Imaging..

[4] Heute D, Kostron H, von Guggenberg E, Ingorokva S, Gabriel M, Dobrozemsky G (2010). Response of recurrent high-grade glioma to treatment with (90)Y-DOTATOC. J Nucl Med..

[5] Reubi JC, Lang W, Maurer R, Koper JW, Lamberts SW (1987). Distribution and biochemical characterization of somatostatin receptors in tumors of the human central nervous system. Cancer Res..

[6] Mawrin C, Schulz S, Pauli SU, Treuheit T, Diete S, Dietzmann K (2004). Differential expression of sst1, sst2A, and sst3 somatostatin receptor proteins in low-grade and high-grade astrocytomas. J Neuropathol Exp Neurol..

[7] Schmidt M, Scheidhauer K, Luyken C, Voth E, Hildebrandt G, Klug N (1998). Somatostatin receptor imaging in intracranial tumours. Eur J Nucl Med..

[8] Breeman WA, de Blois E, Sze Chan H, Konijnenberg M, Kwekkeboom DJ, Krenning EP (2011). (68)Ga-labeled DOTA-peptides and (68)Ga-labeled radiopharmaceuticals for positron emission tomography: current status of research, clinical applications, and future perspectives. Semin Nucl Med.

[9] Weller M, Pfister SM, Wick W, Hegi ME, Reifenberger G, Stupp R (2013). Molecular neuro-oncology in clinical practice: a new horizon. Lancet Oncol..

[10] Gutman DA, Cooper LA, Hwang SN, Holder CA, Gao J, Aurora TD (2013). MR imaging predictors of molecular profile and survival: multi-institutional study of the TCGA glioblastoma data set. Radiology..

[11] Weller M, Stupp R, Hegi ME, van den Bent M, Tonn JC, Sanson M (2012). Personalized care in neuro-oncology coming of age: why we need MGMT and 1p/19q testing for malignant glioma patients in clinical practice. Neuro Oncol..

[12] Belosi F, Cicoria G, Lodi F, Malizia C, Fanti S, Boschi S (2013). Generator breakthrough and radionuclidic purification in automated synthesis of 68Ga-DOTANOC. Curr Radiopharm..

[13] Logan J (2000). Graphical analysis of PET data applied to reversible and irreversible tracers. Nucl Med Biol..

[14] Tuononen K, Tynninen O, Sarhadi VK, Tyybakinoja A, Lindlof M, Antikainen M (2012). The hypermethylation of the O6-methylguanine-DNA methyltransferase gene promoter in gliomas - correlation with array comparative genome hybridization results and IDH1 mutation. Genes Chromosomes Cancer..

[15] Ålgars A, Lintunen M, Carpen O, Ristamäki R, Sundström J (2011). EGFR gene copy number assessment from areas with highest EGFR expression predicts response to anti-EGFR therapy in colorectal cancer. Br J Cancer..

[16] Haldemann AR, Rosler H, Barth A, Waser B, Geiger L, Godoy N (1995). Somatostatin receptor scintigraphy in central nervous system tumors: role of blood–brain barrier permeability. J Nucl Med..

[17] Hanscheid H, Sweeney RA, Flentje M, Buck AK, Lohr M, Samnick S (2012). PET SUV correlates with radionuclide uptake in peptide receptor therapy in meningioma. Eur J Nucl Med Mol Imaging..

[18] Horbinski C (2013). What do we know about IDH1/2 mutations so far, and how do we use it?. Acta Neuropathol..

[19] Wick W, Meisner C, Hentschel B, Platten M, Schilling A, Wiestler B (2013). Prognostic or predictive value of MGMT promoter methylation in gliomas depends on IDH1 mutation. Neurology..

[20] Cervera P, Videau C, Viollet C, Petrucci C, Lacombe J, Winsky-Sommerer R (2002). Comparison of somatostatin receptor expression in human gliomas and medulloblastomas. J Neuroendocrinol..

[21] Korner M, Waser B, Schonbrunn A, Perren A, Reubi JC (2012). Somatostatin receptor subtype 2A immunohistochemistry using a new monoclonal antibody selects tumors suitable for in vivo somatostatin receptor targeting. Am J Surg Pathol..

[22] Smith JS, Perry A, Borell TJ, Lee HK, O’Fallon J, Hosek SM (2000). Alterations of chromosome arms 1p and 19q as predictors of survival in oligodendrogliomas, astrocytomas, and mixed oligoastrocytomas. J Clin Oncol..

[23] Okuwaki K, Kida M, Mikami T, Yamauchi H, Imaizumi H, Miyazawa S (2013). Clinicopathologic characteristics of pancreatic neuroendocrine tumors and relation of somatostatin receptor type 2A to outcomes. Cancer..

[24] Raggi CC, Maggi M, Renzi D, Calabro A, Bagnoni ML, Scaruffi P (2000). Quantitative determination of sst2 gene expression in neuroblastoma tumor predicts patient outcome. J Clin Endocrinol Metab..

[25] Benali N, Cordelier P, Calise D, Pages P, Rochaix P, Nagy A (2000). Inhibition of growth and metastatic progression of pancreatic carcinoma in hamster after somatostatin receptor subtype 2 (sst2) gene expression and administration of cytotoxic somatostatin analog AN-238. Proc Natl Acad Sci U S A..

[26] Vernejoul F, Faure P, Benali N, Calise D, Tiraby G, Pradayrol L (2002). Antitumor effect of in vivo somatostatin receptor subtype 2 gene transfer in primary and metastatic pancreatic cancer models. Cancer Res..

[27] Wild D, Schmitt JS, Ginj M, Macke HR, Bernard BF, Krenning E (2003). DOTA-NOC, a high-affinity ligand of somatostatin receptor subtypes 2, 3 and 5 for labelling with various radiometals. Eur J Nucl Med Mol Imaging..

